# Basolateral Endocytic Recycling Requires RAB-10 and AMPH-1 Mediated Recruitment of RAB-5 GAP TBC-2 to Endosomes

**DOI:** 10.1371/journal.pgen.1005514

**Published:** 2015-09-22

**Authors:** Ou Liu, Barth D. Grant

**Affiliations:** Department of Molecular Biology and Biochemistry, Rutgers University, Piscataway, New Jersey, United States of America; National Heart, Lung, and Blood Institute, UNITED STATES

## Abstract

The small GTPase RAB-5/Rab5 is a master regulator of the early endosome, required for a myriad of coordinated activities, including the degradation and recycling of internalized cargo. Here we focused on the recycling function of the early endosome and the regulation of RAB-5 by GAP protein TBC-2 in the basolateral *C*. *elegans* intestine. We demonstrate that downstream basolateral recycling regulators, GTPase RAB-10/Rab10 and BAR domain protein AMPH-1/Amphiphysin, bind to TBC-2 and help to recruit it to endosomes. In the absence of RAB-10 or AMPH-1 binding to TBC-2, RAB-5 membrane association is abnormally high and recycling cargo is trapped in early endosomes. Furthermore, the loss of TBC-2 or AMPH-1 leads to abnormally high spatial overlap of RAB-5 and RAB-10. Taken together our results indicate that RAB-10 and AMPH-1 mediated down-regulation of RAB-5 is an important step in recycling, required for cargo exit from early endosomes and regulation of early endosome–recycling endosome interactions.

## Author Summary

When cargo is internalized from the cell surface by endocytosis, it enters a series of intracellular organelles called endosomes. Endosomes sort cargo, such that some cargos are sent to the lysosome for degradation, while others are recycled to the plasma membrane. Small GTPase proteins of the Rabs family are master regulators of endosomes, functioning by acting as molecular switches. As cargo moves through the endosomal system, it must pass from the domain controlled by one Rab-GTPase to the domain controlled by another. Little is known about how transitions along the recycling pathway are controlled. Here we analyze a group of protein interactions that act along the early-to-recycling pathway. Our work shows that RAB-5 deactivation mediated by TBC-2 and its recruiters RAB-10 and AMPH-1 is important for cargo recycling. This work provides mechanistic insight into how Rab proteins controlling different steps of trafficking interact during endocytic recycling.

## Introduction

Endocytic recycling, the return of proteins and lipids from endosomes to the plasma membrane, plays a key role in many essential cellular processes including nutrient uptake, cell migration, cytokinesis, synaptic plasticity, immune response, and growth factor receptor modulation [1]. In polarized epithelial cells an additional layer of complexity in the endocytic pathway contributes to formation and/or maintenance of the specialized apical and basolateral domains [2,3]. Both the apical and basolateral membranes deliver cargo to early endosomes, often referred to as apical early endosomes and basolateral early endosomes [3–5]. Basolaterally derived and apically derived cargo can reach common recycling endosomes, from which cargo is sorted for delivery to the basolateral plasma membrane or to apical recycling endosomes [3–5]. The apical recycling endosomes are thought to send their cargo to the apical plasma membrane. Small GTPases of the Rab superfamily play key roles in membrane transport, with at least one Rab protein regulating each transport step. In polarized epithelial cells Rab11 is primarily associated with the apical recycling endosomes and is thought to function in the transport of cargo from the apical recycling endosomes to the plasma membrane [3,6,7]. Rab8 has also been implicated in apical recycling in the intestinal epithelia of mice and worms [8].

Our attention was first brought to bear on the basolateral recycling pathway of *C*. *elegans* intestinal epithelia because of the accumulation of grossly enlarged basolateral vesicles in mutants lacking the recycling regulator RME-1/EHD [9]. In the case of *rme-1* mutants, these enlarged vesicles accumulated recycling cargo and were positive for the endosomal recycling regulator ARF-6, but lacked early endosome marker RAB-5, suggesting that RME-1 functions at a late recycling step [9–11]. Pulse-chase data in mammalian cells showed that loss of mRme-1/EHD1 likewise resulted in a block in recycling endosome to plasma membrane transport [12,13]. Similarly *rab-10* mutants first caught our attention because they displayed enlarged basolateral vesicles in the *C*. *elegans* intestine that accumulated recycling cargo [10]. However, in this case the enlarged endosomes were positive for RAB-5, indicating an earlier block in basolateral recycling, at the level of early endosome to recycling endosome transport [10]. We extended this work, identifying two RAB-10 effectors that function with RAB-10 in basolateral recycling, EHBP-1 and CNT-1 [11,14]. EHBP-1 strongly labeled the tubular elements of the recycling pathway, was required for strong RAB-10 endosomal recruitment, and may link endosomes to the cytoskeleton [14,15]. CNT-1/ACAP is recruited to endosomes by RAB-10 and regulates the activity of ARF-6, acting as part of a small GTPase regulatory loop [11]. In turn ARF-6 regulates PI5-kinase, controlling PI(4,5)P2 levels on basolateral recycling endosomes, and the recruitment of downstream PI(4,5)P2 lipid binding proteins such as RME-1 [11,16].

*C*. *elegans* RAB-10 and human Rab10 are now known to contribute a wide range of endocytic recycling pathways. Like its *C*. *elegans* homolog, mammalian Rab10 functions in basolateral recycling in polarized MDCK cells, where Rab10 localized to basolateral sorting endosomes and the common recycling endosome [17]. *C*. *elegans* RAB-10 is also required for the postsynaptic recycling of glutamate receptors in interneurons [18], and dense-core vesicle secretion of neuropeptides by motor neurons [19]. Mammalian Rab10 is required for toll-like receptor 4 recycling in activated macrophages [20], membrane insertion of plasmalemmal precursor vesicles during neuronal polarization and axonal growth [21,22], and insulin-stimulated glucose transporter recycling in adipocytes [23]. Expression of human Rab10 in the *C*. *elegans* intestine rescues *rab-10* mutant defects, indicating a high degree of functional conservation, suggesting that further elucidating RAB-10 function in *C*. *elegans* will provide mechanistic insight into RAB-10/Rab10 function in many or all of these related processes [10].

Countercurrent cascades of Rab GEFs and Rab GAPs have been proposed to mediate Rab conversion, a process by which Rab proteins interact, helping to establish vectorial transport of cargo along membrane trafficking pathways [24]. In such cascades early acting Rab-GTPases recruit effectors that activate later acting Rab-GTPases, and in turn later acting Rab-GTPases recruit effectors that inactivate early acting Rab-GTPases [24]. However little is known of how such cascades contribute to endocytic recycling. Here we show that RAB-10 recruits the RAB-5 GTPase-activating-protein TBC-2 to endosomes in a step necessary for early endosome to recycling endosome transport. This negative feedback from RAB-10 to RAB-5 is required for the exit of recycling cargo from early endosomes. We also show that the BAR-domain protein AMPH-1 is a binding partner of TBC-2 important for recruitment of TBC-2 to endosomes, functioning as part of the transition of cargo from the early to recycling endosome compartments.

## Results

### RAB-5 GAP TBC-2 is a RAB-10 binding partner

**Fig 1:**
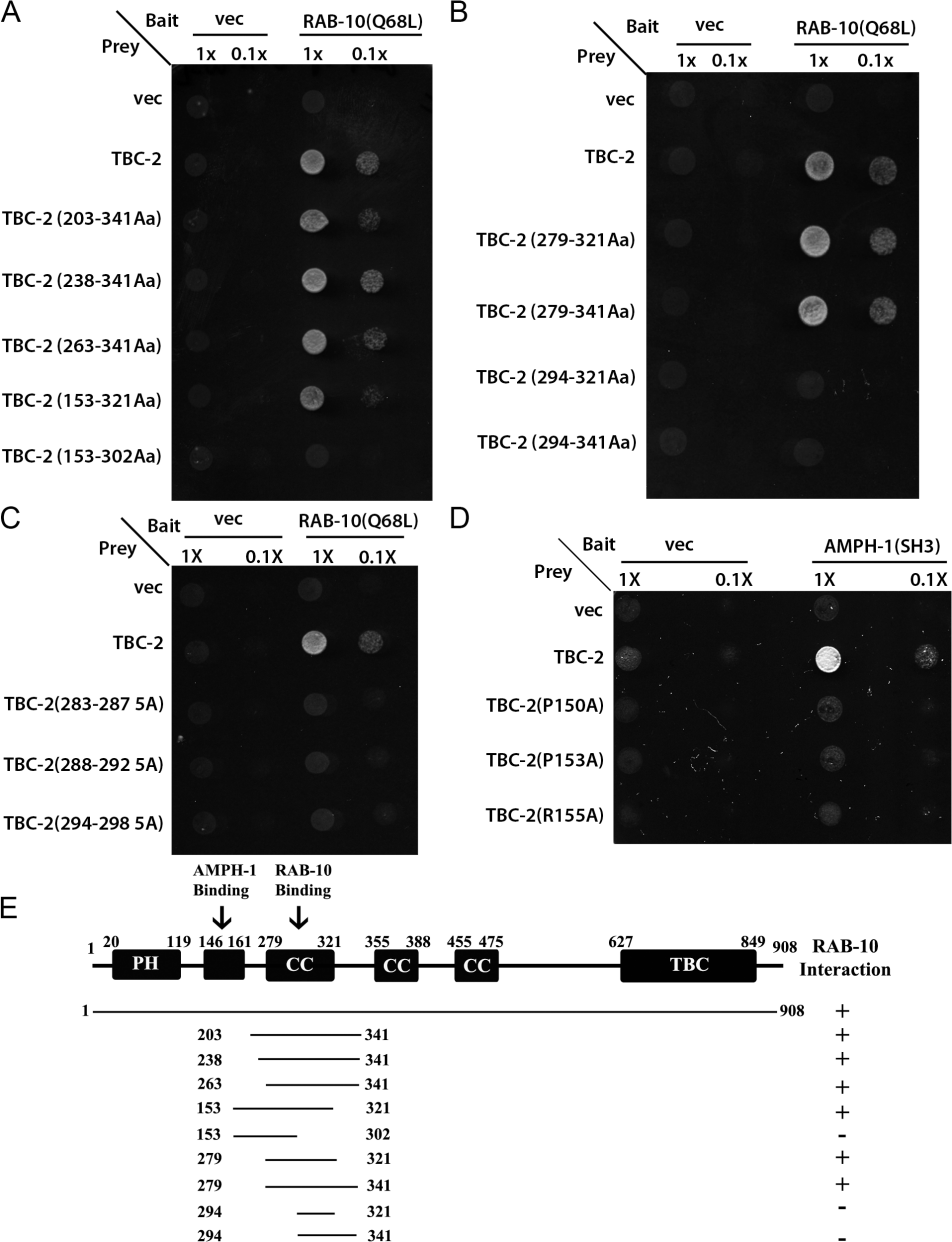
TBC-2 interacts physically with RAB-10 and AMPH-1. (A) and (B) The interaction between TBC-2 and RAB-10(Q68L) requires a segment of TBC-2(AA 279–321) containing a predicted coiled-coil domain. RAB-10(Q68L) was expressed in a yeast reporter strain as a fusion to the DNA-binding domain of LexA (bait). Different truncations of TBC-2 were expressed in the same yeast cells as fusions to the B42 transcriptional activation domain (prey). Interaction between bait and prey was assayed by complementation of leucine auxotrophy (LEU2 growth assay). Colonies were diluted in liquid and spotted on solid growth medium directly (1X) or after further dilution (0.1X). (C) Alanine substitutions within the critical RAB-10-binding sequence of TBC-2 (positions aa283-287, aa288-292, aa294-298) abolished the interaction between TBC-2 and RAB-10(Q68L). (D) Full-length TBC-2 interacts with AMPH-1. Mutation of key residues (prolines P150 or P153, or arginine R155) within TBC-2 (aa146-160), the predicted consensus sequence for AMPH-1 SH3-domain-binding, into alanine via site-directed mutagenesis disrupted the interaction between TBC-2 and the SH3 domain of AMPH-1. AMPH-1 SH3 domain was expressed as bait. Full-length and mutated forms of TBC-2 were expressed as prey. Their interactions were detected using the same two-hybrid assay as described in (A) and (B). (E) Schematic representation of TBC-2 domains and the truncated fragments of TBC-2 used in the Y2H analysis. Protein domains are displayed as dark boxes above the protein sequences (represented by dark lines). Amino acid numbers are indicated.

We have previously reported several proteins that function with RAB-10 in basolateral recycling in the *C*. *elegans* intestine, some of which we first identified via a yeast two-hybrid screen that used a predicted constitutively GTP-bound form of RAB-10(Q68L) as bait [14]. In this same yeast two-hybrid screen we also identified a RAB-10(Q68L) interacting clone encoding full-length TBC-2, a GAP for the earlier acting endosomal GTPase RAB-5 [13,22,25]. The interaction between RAB-10(Q68L) and TBC-2 was positive in both Leu2 and β-galactosidase expression assays (Fig 1A). Using successive truncations of TBC-2 we narrowed the RAB-10 binding site to a 42 amino acid region of TBC-2 (amino acids 279–321) (Fig 1A, 1B and 1E). We noted several runs of highly charged residues in this region, which may represent hydrophilic surface features, and tested their importance for binding to RAB-10 in groups of 5 by alanine scanning. The interaction was abolished when alanine substitutions were imposed at TBC-2 positions aa283-287, aa288-292, and aa294-298 (Fig 1C). These mutations could disrupt binding of TBC-2 to RAB-10 by directly removing surface features involved in the binding interface, or could disrupt the local structure of this region of TBC-2 interfering with binding. Taken together, our results indicate the presence of a predicted coiled-coil domain of TBC-2 that interacts with RAB-10, a key regulator of the basolateral endocytic recycling process. Since TBC-2 is known to act as a GAP for early endosome master regulator RAB-5, these results suggest a negative feedback loop from RAB-10 to RAB-5, potentially acting as part of a RAB cascade in the basolateral recycling pathway.

### TBC-2 is highly enriched on RAB-10-positive endosomes

**Fig 2:**
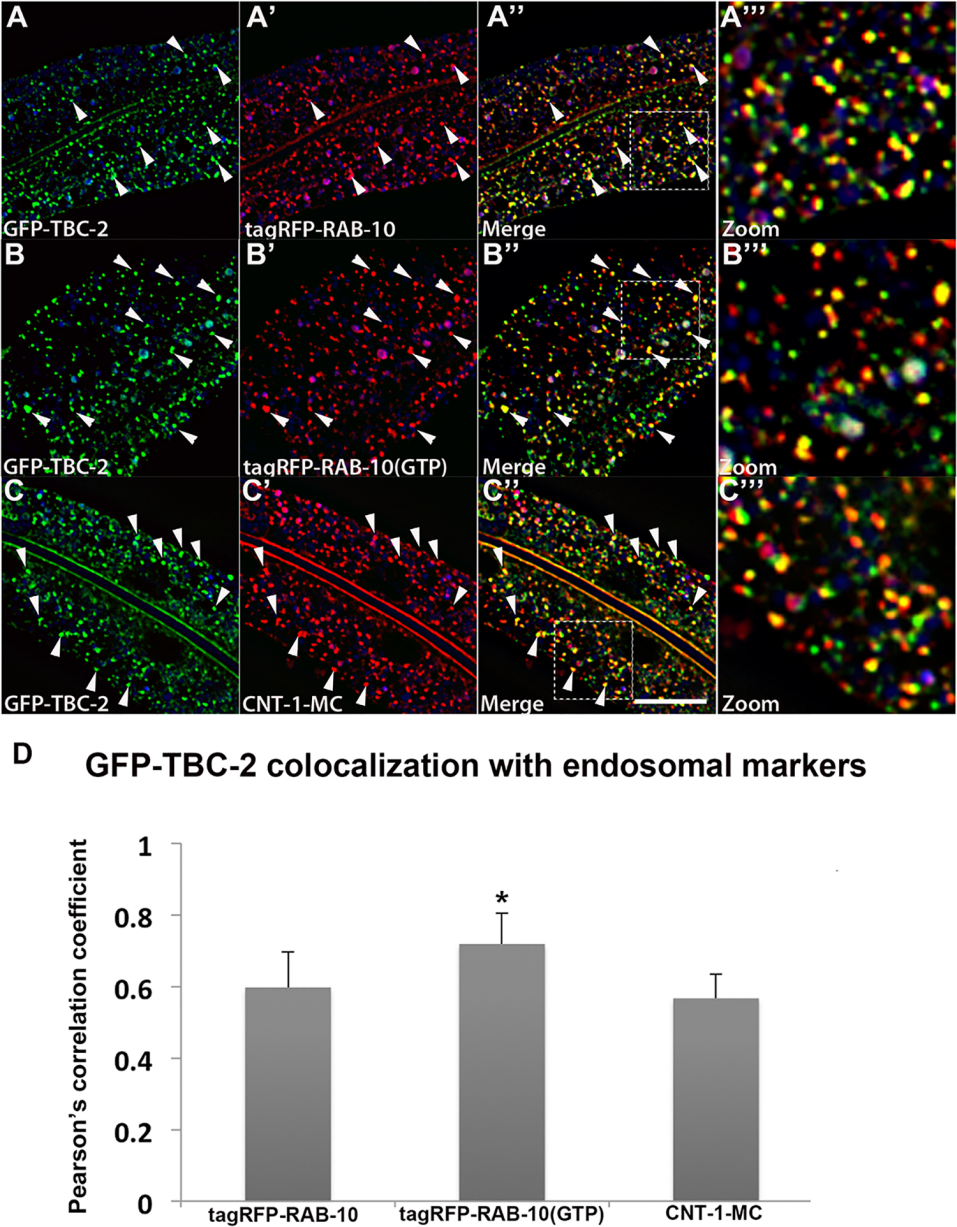
TBC-2 colocalizes with RAB-10 and CNT-1 on endosomes. All images are from deconvolved 3D confocal image stacks acquired in intact living animals expressing GFP- and RFP-tagged proteins specifically in intestinal epithelial cells. (A-A'') GFP-TBC-2 colocalizes well with tagRFP-RAB-10. Arrowheads indicate endosomes labeled by both GFP-TBC-2 and tagRFP-RAB-10. (A‴) Magnified image of A'' is designated by rectangular outline. (B-B'') GFP-TBC-2 colocalizes extensively with tagRFP-RAB-10(Q68L), a predicted constitutively active form of RAB-10. Arrowheads indicate endosomes labeled by both GFP-TBC-2 and tagRFP-RAB-10(Q68L). (B‴) Magnified image of B'' is designated by rectangular outline. (C-C'') GFP-TBC-2 colocalizes well with CNT-1-MC. Arrowheads indicate endosomes labeled by both GFP-TBC-2 and CNT-1-MC. (C‴) Magnified image of C'' is designated by rectangular outline. In each image autofluorescent lysosome-like organelles appear in blue in all three channels, whereas GFP appears only in the green channel and RFP shows up only in the red channel. Signals observed in the green or red channels that do not overlap with signals in the blue channel are considered bona fide GFP or RFP signals respectively. (Scale bar: 10 μm) (D) Pearson's correlation coefficient for colocalization of GFP-TBC-2 with tagRFP-RAB-10, tagRFP-RAB-10(Q68L), and CNT-1-MC. n = 6. Error bars represent SEM. *P<0.05(student's t test).

Intestinally expressed GFP-tagged TBC-2 labels abundant cytoplasmic puncta with the typical size and shape of endosomes (~250–500 nm diameter). If TBC-2 is a physiologically relevant binding partner for RAB-10, we would expect to find RAB-10 and TBC-2 on the same population of endosomes *in vivo*. Previous qualitative work indicated some localization of TBC-2 to early and late endosomes, but the extent of localization, and its relationship to recycling endosomes, remained unclear [22,25]. To quantitatively test the subcellular localization of TBC-2 we conducted a series of co-localization studies in the intestinal epithelial cells where RAB-10 is known to function, using a set of previously established RFP markers for RAB-10 and a variety of endocytic compartments. The degree of colocalization was measured using Pearson’s correlation coefficient, a statistical measure of the degree of linear dependence of the GFP and RFP signals [26]. Consistent with our binding data, we detected the greatest correlation coefficient of GFP-TBC-2 with RFP-tagged RAB-10(+) and constitutively active RFP-RAB-10(Q68L) (Fig 2A–2A‴ and 2B–2B‴ and Fig 2D). The greater degree of correlation of TBC-2 signal with RAB-10(Q68L) signal is consistent with a model where RAB-10 helps to recruit TBC-2 onto endosomes. GFP-TBC-2 signal also correlated very well with a previously characterized RAB-10 effector, CNT-1 (CNT-1-mCherry) (Fig 2C–2C‴ and Fig 2D), which is also required for the recycling process [11]. These results are consistent with TBC-2 acting with RAB-10 and CNT-1 in the basolateral endocytic recycling.

GFP-TBC-2 signals also showed lesser, but significant, correlations with early endosomal marker tagRFP-RAB-5 (S2A–S2A'' and S2D Fig) and late endosomal marker tagRFP-RAB-7 (S2B–S2B'' Fig and S2D Fig). We also noted that the GFP-TBC-2 signal displays hardly any correlation with that of EHBP-1-mCherry, another RAB-10 interacting protein that labels tubular aspects of the basolateral recycling endosome network (S2C–S2C'' Fig). Collectively, our results indicate that TBC-2 is enriched on a subpopulation of endosomes, where it could function with RAB-10 and RAB-5 to confer effective transport of cargo during the endocytic recycling process.

### RAB-10 is required for the endosomal recruitment of TBC-2 in the intestinal epithelium

**Fig 3:**
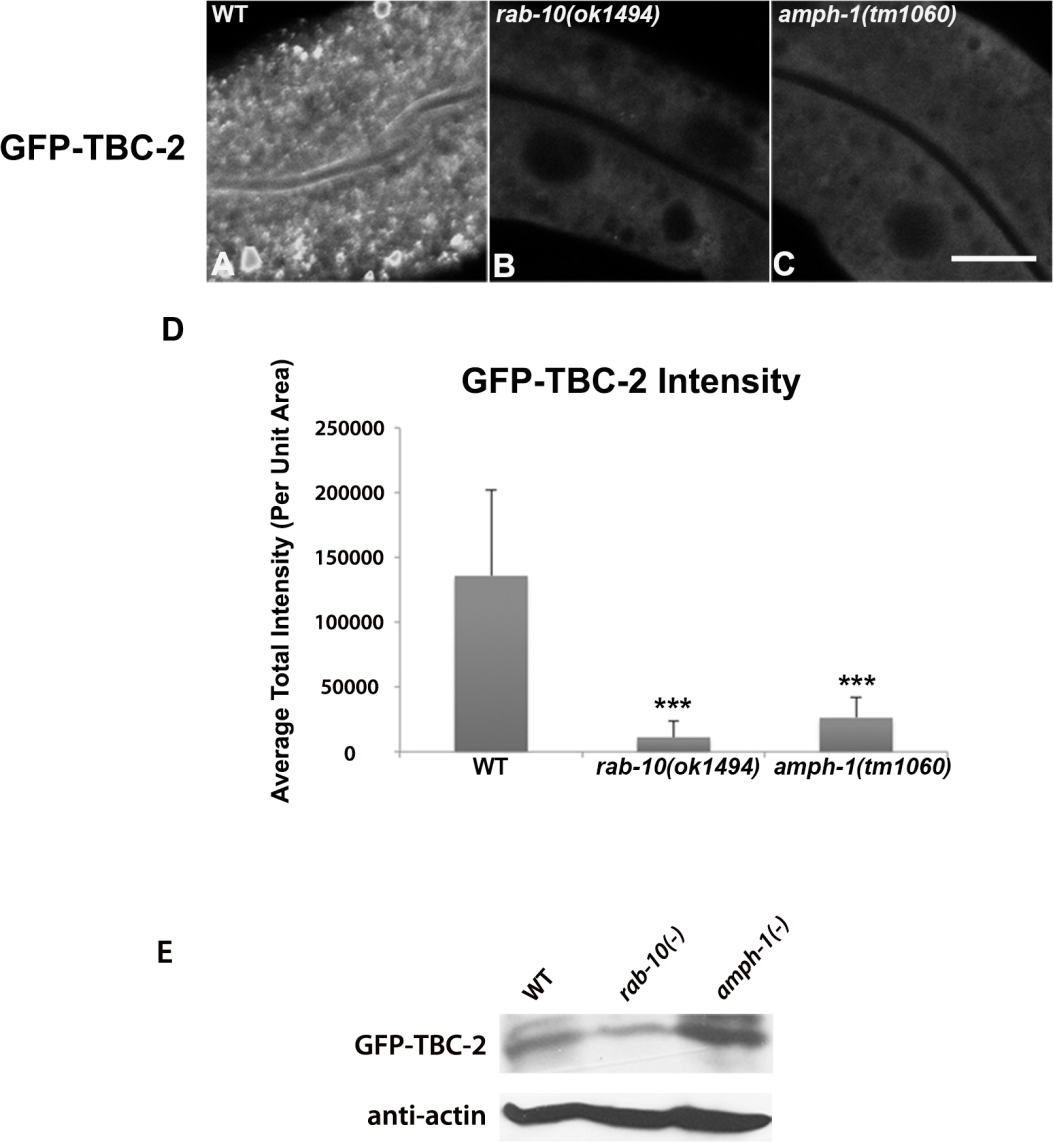
RAB-10 and AMPH-1 contribute to the endosome recruitment of TBC-2. All images were collected from living intact adult animals expressing intestine-specific transgenes. Representative confocal images of GFP-TBC-2 in wild-type (A), *rab-10(ok1494)* mutant (B), and *amph-1(tm1060)* mutant (C) backgrounds are shown. The endosomal localization of GFP-TBC-2 is strongly reduced in *rab-10(ok1494)* and *amph-1(tm1060)* mutant backgrounds. (Scale bar: 10 μm) (D) Quantification of the average total intensity of GFP-TBC-2 labeled structures. (n = 18 each, 6 animals of each genotype sampled in three different regions of each intestine.) Error bars represent SEM. ***P<0.001(student's t test). (E) Western Blot analysis of young adult worms expressing GFP-TBC-2 in wild-type, *rab-10(ok1494)* mutant, and *amph-1(tm1060)* mutant backgrounds. Blots with anti-GFP and anti-actin antibodies are shown.

**Fig 4:**
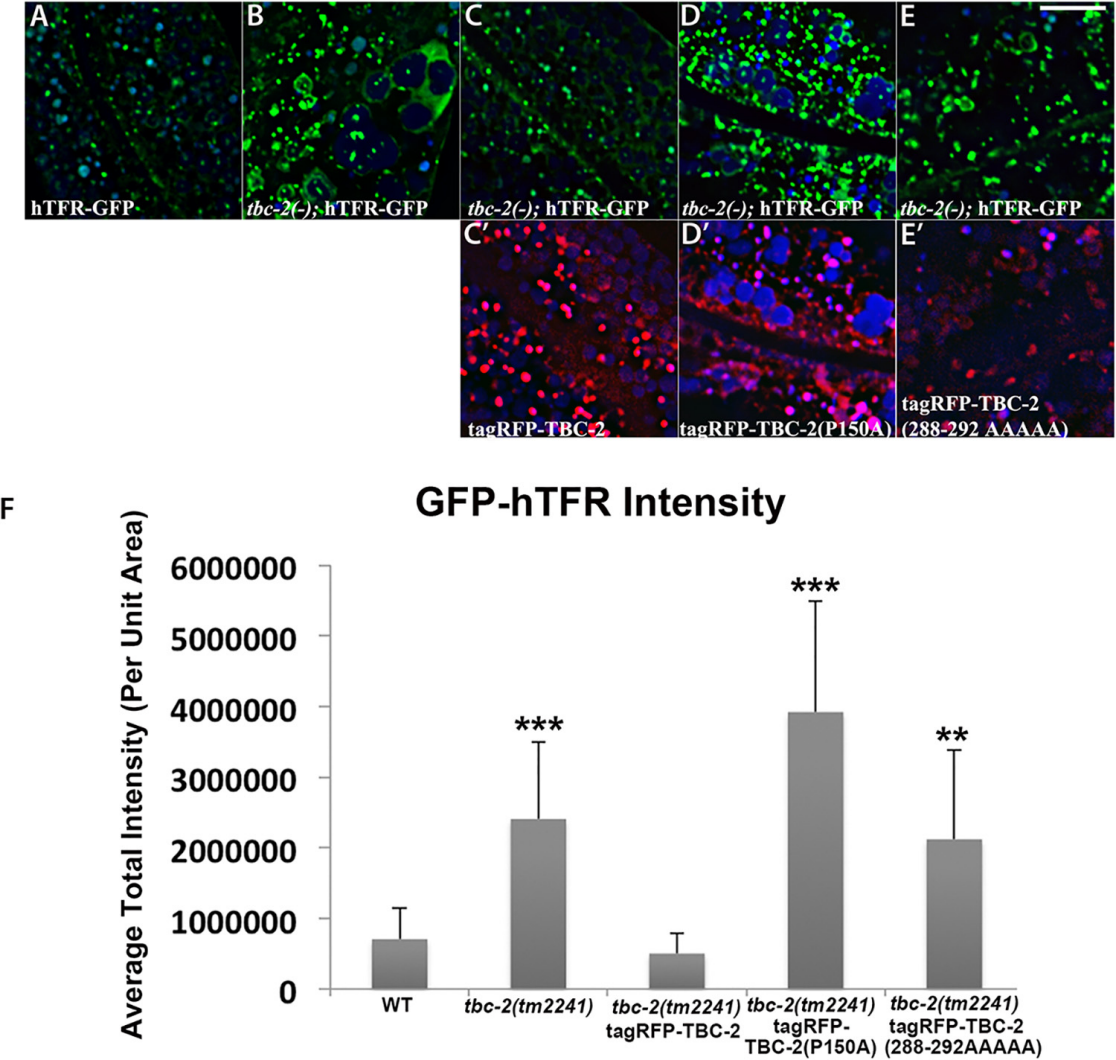
Rescue of the cargo-recycling defect of *tbc-2* mutants requires intact RAB-10 and AMPH-1 interaction sequences. All images are from deconvolved 3D confocal image stacks acquired in intact living animals expressing GFP- and RFP-tagged proteins. (A-E) Representative confocal images of the worm intestine expressing a GFP-tagged recycling cargo protein, the human transferrin receptor (hTFR-GFP). Loss of TBC-2 caused accumulation of hTFR-GFP on abnormally enlarged endosomal structures. The *tbc-2* mutant phenotype in cargo recycling is rescued by expression of RFP-tagged full-length TBC-2 in the worm intestine. However, expression of mutant forms of RFP-tagged TBC-2 defective either in AMPH-1-binding (TBC-2[P150A]) or in RAB-10-binding (TBC-2[288–292 AAAAA]) in the worm intestine failed to rescue the *tbc-2* mutant phenotype. (C') Confocal image of the worm intestine expressing RFP-tagged wild-type TBC-2. (D') Confocal image of the worm intestine expressing RFP-tagged mutant form of TBC-2 with alanine substitution at proline P150. (E') Confocal image of the worm intestine expressing RFP-tagged mutant form of TBC-2 with five alanines replacing amino acids 288–292. (Scale bar: 10 μm) (F) Quantification of the average total intensity of hTFR-GFP. In each image autofluorescent lysosome-like organelles appears in blue in all three channels, whereas GFP appears only in the green channel and RFP shows up only in the red channel. Signals observed in the green or red channels that do not overlap with signals in the blue channel are considered bona fide GFP or RFP signals respectively. (n = 18 each, 6 animals of each genotype sampled in three different regions of each intestine.) Error bars represent SEM. **P<0.01, ***P<0.001(student's t test).

To further test the idea that an interaction with RAB-10 is important for TBC-2 function *in vivo*, we examined the effect of a *rab-10* loss-of-function mutant on the endosomal localization of GFP-TBC-2 in the intestinal epithelia. In the *rab-10* mutant background GFP-TBC-2 became very diffusive, losing its typical punctate endosomal localization, indicating a requirement for RAB-10 in TBC-2 endosomal recruitment (Fig 3A and 3B). Western blot analysis also showed that GFP-TBC-2 levels are reduced in *rab-10* mutants, suggesting that TBC-2 is less stable in the absence of RAB-10 (Fig 3E). We extended this analysis further, testing a form of TBC-2 impaired for RAB-10 binding (QRNNE 288–292 AAAAA) for function *in vivo*. In previous work we showed that TBC-2 is required for the normal recycling of model cargo hTfR-GFP (human transferrin receptor–GFP)[27]. In the absence of TBC-2, hTfR-GFP accumulates in enlarged intracellular structures (Fig 4A, 4B and 4F). While expression of full length wild-type TBC-2 efficiently rescued the localization of hTfR-GFP in a *tbc-2* null mutant background (Fig 4A–4C and 4F), we found that expression of the interaction defective form of TBC-2 failed to rescue the localization of hTfR-GFP in a *tbc-2* null mutant background (Fig 4E and 4F).

### Physical interaction between TBC-2 and AMPH-1

In many cases peripheral membrane proteins of the endosome require multiple protein and/or lipid interactions to direct their localization. Recent work using phage-display to identify the binding preferences of all *C*. *elegans* SH3 domains suggested a link between TBC-2 and AMPH-1, a BAR-domain and SH3-domain protein that is the only *C*. *elegans* member of the Amphiphysin/BIN1 protein family [28,29]. TBC-2 amino acid sequence 146–160 was identified as the fourth best match for the AMPH-1 SH3-domain binding consensus in the entire predicted *C*. *elegans* proteome [28]. Previous work from our laboratory has shown that AMPH-1 participates in the basolateral recycling pathway [28,29]. Thus we sought to further examine this potential interaction. We detected interaction of full-length TBC-2 with the AMPH-1 SH3 domain in a yeast 2-hybrid assay (Fig 1D). Importantly, the interaction was abolished when key residues in the consensus sequence, prolines P150 or P153, or arginine R155, were mutated to alanine (Fig 1D and 1E). Despite losing their ability to interact with AMPH-1, the P150A, P153A, and R155A mutant forms of TBC-2 protein retained the ability to interact with RAB-10(Q68L) in the same two-hybrid assay, indicating that the mutant forms of TBC-2 were stable (S1A Fig). We conclude that the AMPH-1 SH3 domain has the potential to bind to the predicted target sequence in TBC-2 (Fig 1D and S1A Fig).

### AMPH-1 contributes to the endosomal recruitment of TBC-2

If an interaction between AMPH-1 and TBC-2 is important *in vivo*, we might expect to observe a change in TBC-2 localization in an *amph-1* mutant background. Indeed, when we examined the subcellular localization of intestinally expressed GFP-TBC-2 in an *amph-1* deletion mutant, we found that the normal punctate endosomal distribution of GFP-TBC-2 was severely disrupted (Fig 3A, 3C and 3D). Instead, GFP-TBC-2 appeared quite diffusive in the absence of AMPH-1, indicating that AMPH-1 is important for endosomal recruitment of TBC-2 (Fig 3C). GFP-TBC-2 levels as assayed by western blot were not reduced in *amph-1* mutants (Fig 3E).

We extended this analysis further, testing a form of TBC-2 impaired for AMPH-1 binding (P150A) for function *in vivo*, using the same hTfR-GFP localization assay described above. We found that while expression of full length wild-type TBC-2 efficiently rescued the localization of hTfR-GFP in a *tbc-2* null mutant background (Fig 4A–4C and 4F), the expression of the interaction defective form of TBC-2 failed to rescue the localization of hTfR-GFP in a *tbc-2* null mutant background (Fig 4A, 4B, 4D and 4F). Our results thus indicate that in addition to RAB-10, AMPH-1 also contributes to TBC-2 endosomal recruitment.

### AMPH-1 interacts with RAB-10

We also determined that AMPH-1 can interact with RAB-10 using a GST-pulldown approach with full length GST-AMPH-1 and HA-tagged RAB-10(Q68L) (S3C Fig). Addition of FLAG-TBC-2 to this assay showed that GST-AMPH-1 can pull down TBC-2 and RAB-10 at the same time, but the presence of TBC-2 in the reaction did not appear to increase the pulldown efficiency of RAB-10 (S3C Fig). Colocalization analysis indicated the presence of AMPH-1-GFP and tagRFP-RAB-10 on a significant fraction of the same endosomes, consistent with physiological significance for the AMPH-1/RAB-10 interaction (S3A and S3B Fig). However, loss of RAB-10 did not reduce association of AMPH-1-GFP with membranes (S4A–S4C Fig). Rather in *rab-10* mutants we observed an increase in AMPH-1-GFP puncta and tubule intensity (S4A–S4C Fig). This may be an indirect effect of the increase in endosomal PI(4,5)P2 in *rab-10* mutants that we previously showed occurs in part via another RAB-10 effector CNT-1, an ARF-6 GAP [11]. Alternatively RAB-10 may affect AMPH-1 recruitment or function more directly, perhaps affecting its conformation or interaction with other proteins.

### RAB-5 displays elevated membrane-association in *tbc-2*, *rab-10*, and *amph-1* mutants

If RAB-10 and AMPH-1 contribute to TBC-2 recruitment and function, then loss of RAB-10 or AMPH-1 would be expected to result in abnormally elevated levels of GTP-bound RAB-5. Furthermore, since the Rab protein nucleotide cycle is linked to Rab protein membrane association, an elevated "active" GTP-bound status for RAB-5 should result in an elevated level of membrane-bound RAB-5. This model predicts that in *tbc-2*, *rab-10*, and *amph-1* mutants, where the RAB-5 GAP TBC-2 is either completely missing, or is mislocalized, RAB-5 association with membranes should be increased. Previous work showed that RAB-5 labeled endosomes are enlarged and/or more numerous in *tbc-2* and *rab-10* mutants, consistent with this model [10,25,27]. In our previous work we had assayed RAB-5 labeled early endosome number in *amph-1* mutants and found no significant change [29]. However, in light of the interaction of AMPH-1 with TBC-2, we analyzed additional parameters, and found that RAB-5 puncta intensity is increased in *amph-1* mutants, consistent with elevated RAB-5 membrane association (S5 Fig).

**Fig 5:**
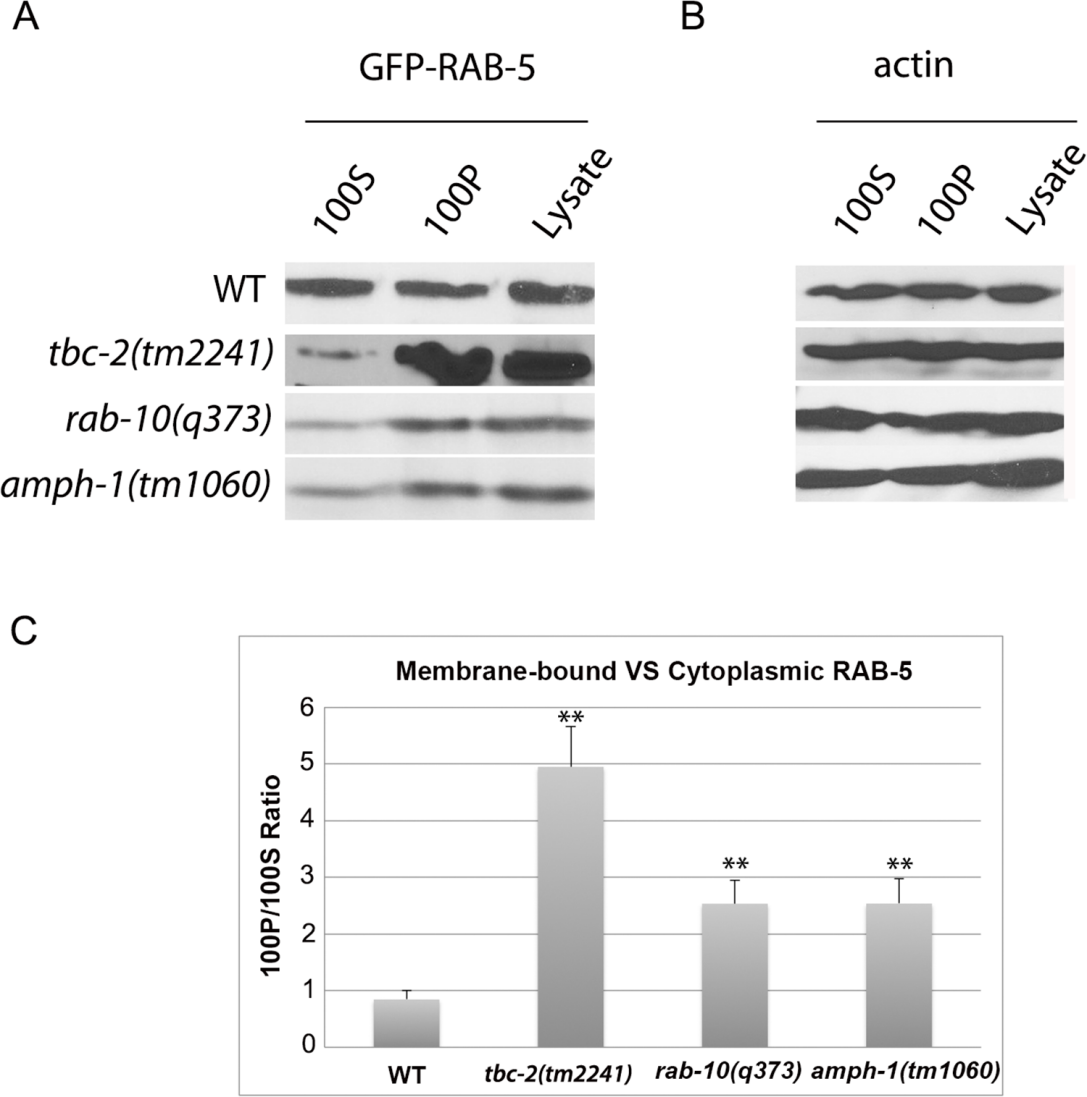
RAB-5 displays elevated membrane-association in *tbc-2*, *rab-10* and *amph-1* mutants. (A) The membrane-to-cytosol ratio of RAB-5 increased in *tbc-2*, *rab-10* and *amph-1* mutants. Post-nuclear worm lysates were subject to centrifugation at 100,000g for 1 hour. 100P corresponds to the pellets and 100S represents the supernatants after the 100,000g centrifugation. (B) Loading control with actin antibody. (C) Quantification of the membrane-to-cytosol ratio (100P/100S) of RAB-5 in appropriate genetic backgrounds. The ratio of membrane-bound VS cytosolic RAB-5 was determined by densitometry. The standard deviations from three independent experiments are shown. **P<0.01 (student's t test).

Endosome size and number can change for a number of reasons, so we extended this analysis to directly measure RAB-5 membrane association biochemically. We separated membranes from cytosol in *C*. *elegans* lysates using ultracentrifugation at 100,000g in the appropriate mutant backgrounds, comparing the amount of intestinally expressed GFP-RAB-5 present in each fraction by Western blot. Consistent with the predictions from this model, we observed an elevation in GFP-RAB-5 membrane-to-cytosol ratio in *tbc-2*, *rab-10*, and *amph-1* mutants (Fig 5A–5C). Loss of RAB-10 or AMPH-1 increased the membrane association of RAB-5 to a lesser extent than that caused by loss of TBC-2, suggesting that some localized TBC-2 remains in *rab-10* and *amph-1* mutants, although endosome localized TBC-2 is difficult to visualize by microscopy in such mutant backgrounds (Fig 5A and Fig 5C). In summary, our data support a role for *rab-10* and *amph-1* in TBC-2 membrane recruitment that is required to complete the RAB-5 nucleotide cycle, removing RAB-5 from membranes. Since RAB-10 and AMPH-1 function in the recycling aspect of endocytic trafficking, these results suggest that removal of RAB-5 from endosomal membranes is an integral part of the recycling process, perhaps linked to cargo transition from early to recycling endosome transport.

### Loss of TBC-2 or AMPH-1 alters the spatial coordination of RAB-5 and RAB-10

**Fig 6:**
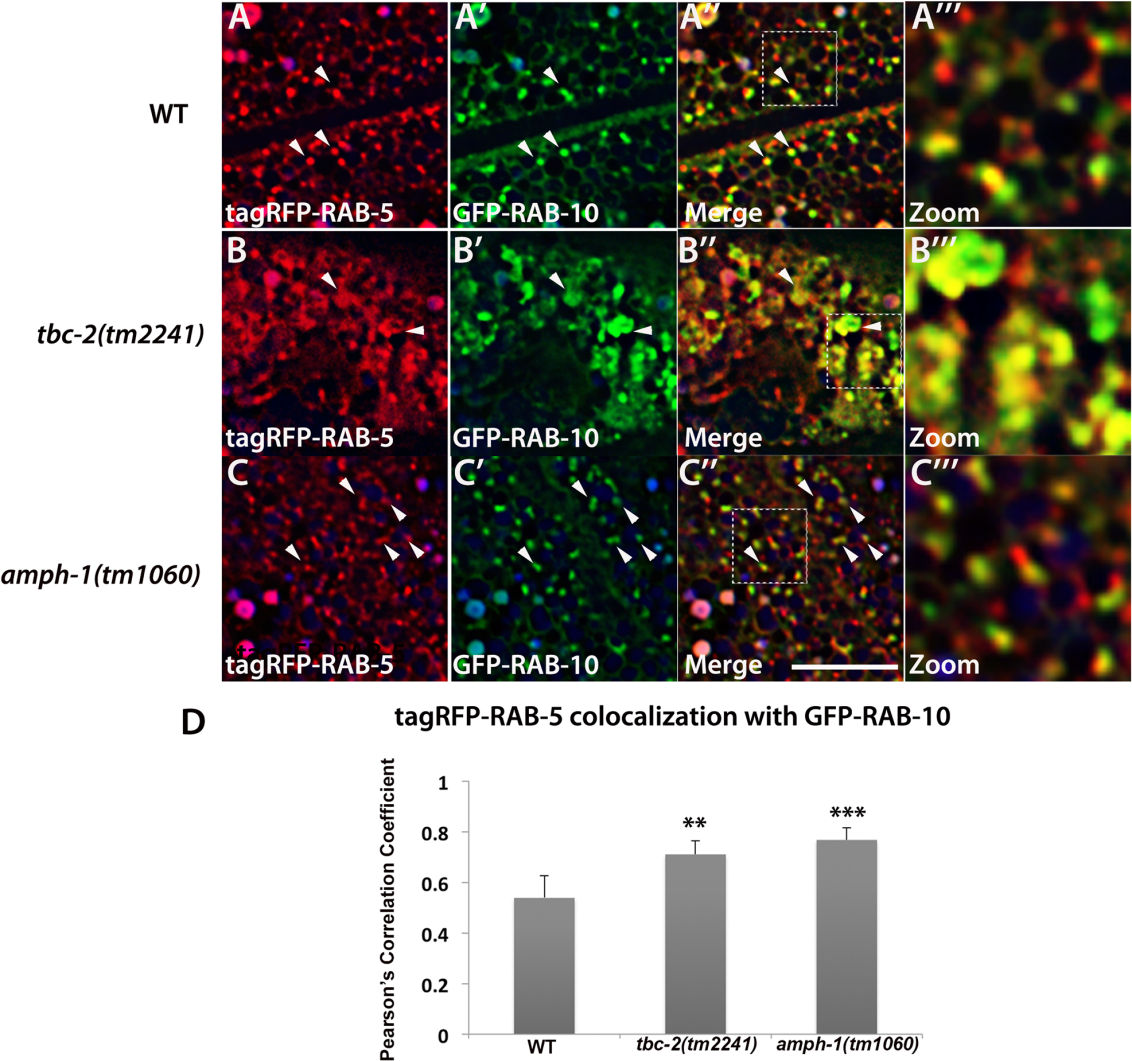
Loss of TBC-2 or AMPH-1 alters the spatial coordination of RAB-5 and RAB-10. All images are from deconvolved 3D confocal image stacks acquired in intact living animals expressing GFP- and RFP-tagged proteins specifically in intestinal epithelial cells. (A-A‴) Under wild-type conditions, tagRFP-RAB-5 and GFP-RAB-10 display partial colocalization (~53%) on punctate endosomal structures. (B-B‴) In *tbc-2 (tm2241)* mutant animals, tagRFP-RAB-5 and GFP-RAB-10 label enlarged pleiomorphic structures and their degree of overlap increased to ~71%. (C-C‴) *amph-1* mutants also displayed increased overlap between tagRFP-RAB-5 and GFP-RAB-10 (~76%) with less severe morphological change than that caused by *tbc-2* mutants. In each image autofluorescent lysosome-like organelles appears in blue in all three channels, whereas GFP appears only in the green channel and RFP shows up only in the red channel. Signals observed in the green or red channels that do not overlap with signals in the blue channel are considered bona fide GFP or RFP signals respectively. (Scale bar: 10 μm) (D) Pearson's correlation coefficient for colocalization of tagRFP-RAB-5 and GFP-RAB-10. n = 6. Error bars represent SEM. **P<0.01, ***P<0.001(student's t test).

Previous work showed that RAB-5 and RAB-10 display significant spatial overlap in the *C*. *elegans* intestine, consistent with functional data indicating that RAB-10 is important for exit of recycling cargo from RAB-5-positive endosomes [10]. To better understand the relationship between RAB-5 and RAB-10, we assayed for changes in their relative colocalization in *tbc-2* and *amph-1* mutants. Similar to previously published results, we found that under wild-type conditions tagRFP-RAB-5 and GFP-RAB-10 both label punctate endosomal structures that partially colocalize (Fig 6A–6A‴ and 6D). We detected dramatic morphological changes for both tagRFP-RAB-5 and GFP-RAB-10 labeled endosomes in a *tbc-2* mutant background. Aside from some remaining punctate structures, in *tbc-2* mutants tagRFP-RAB-5 and GFP-RAB-10 tended to label very large pleiomorphic structures that were never observed in wild-type animals (Fig 6B–6B‴). Quantification of RAB-5 colocalization with RAB-10 showed a significant increase in the correlation of tagRFP-RAB-5 and GFP-RAB-10 signals in *tbc-2* mutants (Fig 6D), with colocalization mostly restricted to the grossly enlarged structures (Fig 6B–6B‴). *amph-1* mutants also displayed a significant increase in the correlation of the tagRFP-RAB-5 and GFP-RAB-10 signals (Fig 6C–6C‴ and 6D), although the morphological size and shape changes were less severe than those in *tbc-2* mutants (Fig 6C–6C‴). Taken together, these data suggest that TBC-2 and AMPH-1 cause recycling defects by altering the normal compartmentalization of RAB-5 and RAB-10 on endosomes.

### Recycling cargo accumulates in RAB-5 labeled endosomes in *tbc-2* and *amph-1* mutants

**Fig 7:**
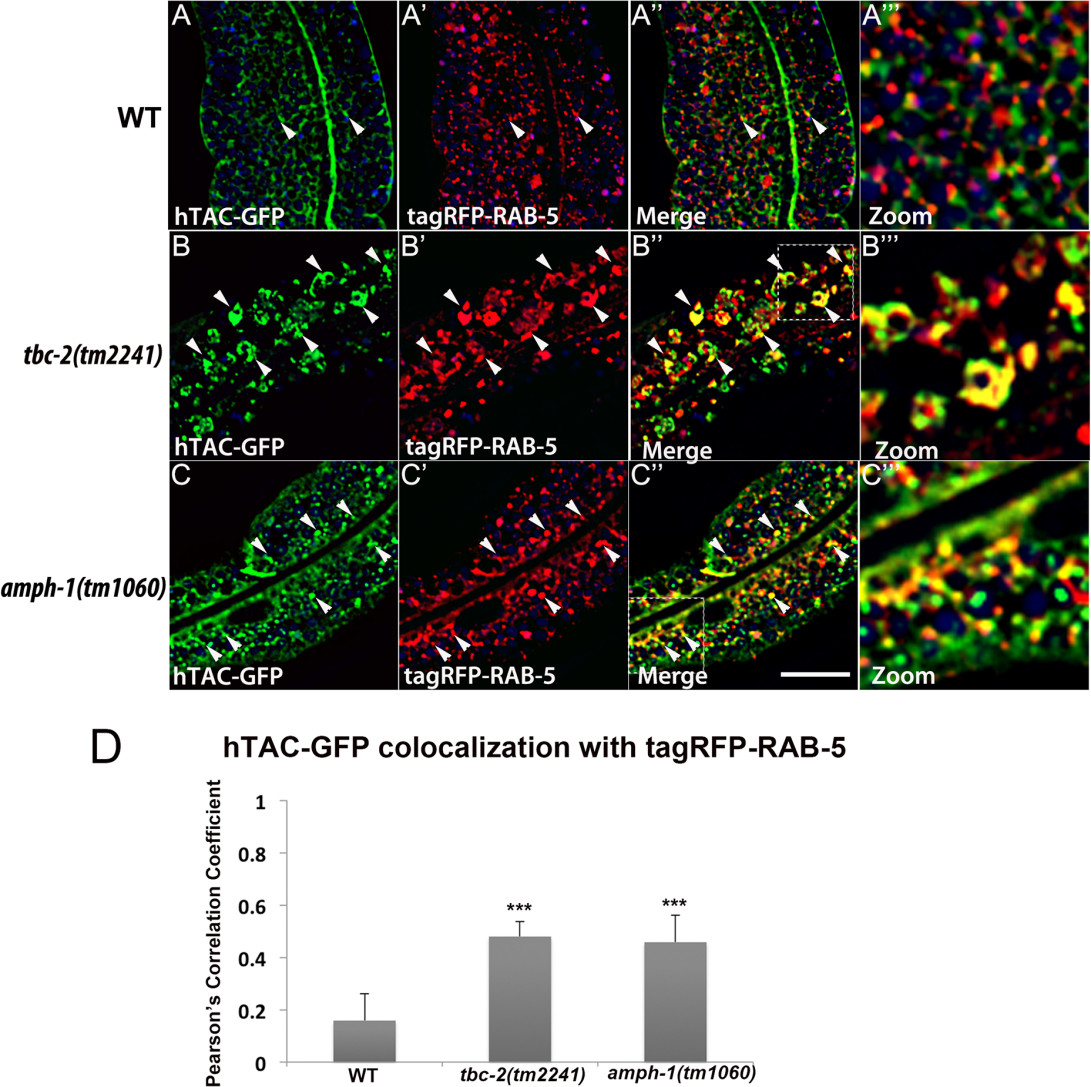
Recycling cargo accumulates in RAB-5 labeled endosomes in *tbc-2* and *amph-1* mutants. All images are from deconvolved 3D confocal image stacks acquired in intact living animals expressing a GFP-tagged recycling cargo protein, the IL-2 receptor alpha-chain (hTAC-GFP) and tagRFP-RAB-5 in wild-type animals (A-A‴), *tbc-2(tm2241)* (B-B‴) and *amph-1(tm1060)* mutants (C-C‴). (A-A‴) In wild-type animals, hTAC-GFP labels both punctate and tubular endosomal structures and has very little overlap with tagRFP-RAB-5. (B-B‴) In *tbc-2* mutants, hTAC-GFP and tagRFP-RAB-5 label abnormally enlarged endosomal structures. There is a striking increase in the degree of overlap between hTAC-GFP and tagRFP-RAB-5 in *tbc-2* mutants in comparison to that in wild-type animals. (C-C‴) In *amph-1* mutants, the degree of colocalization between hTAC-GFP and tagRFP-RAB-5 also increased. In each image autofluorescent lysosome-like organelles appear in blue in all three channels, whereas GFP appears only in the green channel and RFP appears only in the red channel. Signals observed in the green or red channels that do not overlap with signals in the blue channel are considered bona fide GFP or RFP signals respectively. (Scale bar: 10 μm) (D) Pearson's correlation coefficient for colocalization of hTAC-GFP and tagRFP-RAB-5. n = 6. Error bars represent SEM. ***P<0.001(student's t test).

**Fig 8:**
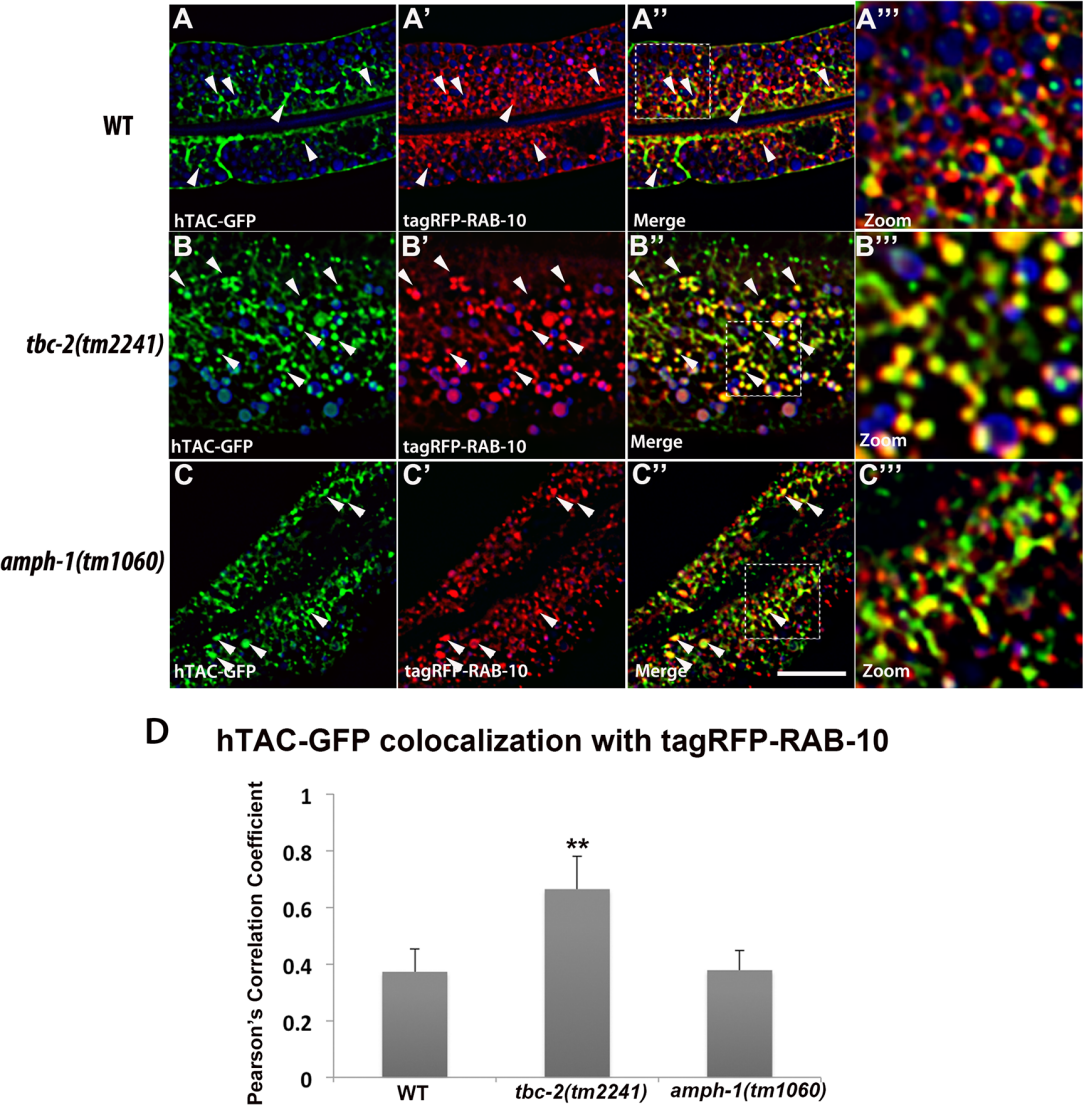
Colocalization of recycling cargo on RAB-10 labeled endosomes. All images are from deconvolved 3D confocal image stacks acquired in intact living animals expressing a GFP-tagged recycling cargo protein, the IL-2 receptor alpha-chain (hTAC-GFP) and tagRFP-RAB-10 in wild-type animals (A-A‴), *tbc-2 (tm2241)* (B-B‴) and *amph-1(tm1060)* mutants (C-C‴). (A-A‴) In wild-type animals, hTAC-GFP and tagRFP-RAB-10 colocalizes partially. (B-B‴) In *tbc-2* mutants, the degree of colocalization between hTAC-GFP and tagRFP-RAB-10 increased and they mainly overlap on punctate endosomal structures. (C-C‴) In *amph-1* mutants, no obvious change in the degree of overlap between hTAC-GFP and tagRFP-RAB-10 was detected in comparison to that in wild-type animals. In each image autofluorescent lysosome-like organelles appears in blue in all three channels, whereas GFP appears only in the green channel and RFP shows up only in the red channel. Signals observed in the green or red channels that do not overlap with signals in the blue channel are considered bona fide GFP or RFP signals respectively. (Scale bar: 10 μm) (D) Pearson's correlation coefficient for colocalization of hTAC-GFP and tagRFP-RAB-10. n = 6. Error bars represent SEM. **P<0.01(student's t test).

Our previous work on RAB-10 function in the intestine showed that RAB-5 labeled endosomes in *rab-10* mutants are grossly enlarged and accumulate an additional model recycling cargo, hTAC-GFP (human TAC, IL-2 receptor alpha chain)[10]. hTAC-GFP strongly labels the tubular aspects of the basolateral recycling pathway at steady state, and depends upon RAB-10, RME-1, and ARF-6 for its recycling [10–11,13]. To better understand the step in recycling transport affected by TBC-2 and AMPH-1 we assayed the relative localization of hTAC-GFP to tagRFP-RAB-5 and tagRFP-RAB-10 in *tbc-2* and *amph-1* mutants. Under wild-type conditions, hTAC-GFP displays little steady-state overlap with tagRFP-RAB-5 (Fig 7A–7A‴). In *tbc-2* mutant animals, the tubular meshwork of hTAC-GFP appears disrupted, with hTAC-GFP mostly found in enlarged endosomes, many of which label for tagRFP-RAB-5 (Fig 7B–7B‴). We measured a striking increase in the degree of colocalization between hTAC-GFP and tagRFP-RAB-5 in *tbc-2* mutants (Fig 7D). In animals lacking AMPH-1, we also detected a significantly larger degree of overlap between hTAC-GFP and tagRFP-RAB-5 in comparison to that of wild-type animals (Fig 7C–7C‴ and 7D). Consistent with our previous reports, we observed partial overlap of hTAC-GFP with tagRFP-RAB-10, mostly restricted to punctate rather than tubular aspects of the hTAC-GFP labeled endosomes (Fig 8A–8A‴). The degree of colocalization between hTAC-GFP and tagRFP-RAB-10 increased mildly in *tbc-2* mutants and was basically unaltered in *amph-1* mutants (Fig 8A–8A‴, 8B–8B‴, 8C–8C‴ and 8D). Taking into account the aforementioned increase in colocalization between RAB-5 and RAB-10 in these mutant backgrounds, these data suggest that most hTAC-GFP in *tbc-2* mutant and in *amph-1* mutant animals is trapped in the early endosome.

## Discussion

Given the continuous flow of proteins and membranes along the endocytic and exocytic pathways, cells face a formidable challenge in achieving accurate intracellular transport of membrane cargo. Such transport is likely to require tight regulation that enforces the directionality of sequential flow between membranous compartments [24]. Rab GTPases serve as master regulators of membrane trafficking by controlling the structural and functional characteristics of intracellular organelles [24]. The ability to switch between the "on" and "off" states through the Rab GTP/GDP cycle empowers Rab proteins to control the spatial and temporal regulation of cargo transport [30]. Rabs interact with a cohort of effector proteins that contribute to a variety of functions, ranging from vesicle tethering, to vesicle budding and movement, and regulating the activation state of other small GTPases [31]. An ordered relay of cargo between sequentially acting compartments is thought to entail coordination of Rab activation states, coordinating changes in organelle maturation and/or allowing distinct compartments to interact at the right time and the right place for cargo transfer [32]. A Rab cascade model has been proposed that likely defines a general principle in membrane transport. This model proposes that an upstream GTP-loaded Rab protein recruits the GEF for the next Rab-GTPase along a transport pathway, activating the downstream Rab. In turn a countercurrent activity is initiated by the downstream GTP-loaded Rab, which recruits the GAP for the upstream Rab to deactivate it [24]. Together these activities are proposed to help enforce unidirectional flow. Such Rab cascades have been proposed for maturation based transport steps, such as the early endosome to late endosome transition, as well as transport steps mediated by small vesicle transport between distinct compartments, such as ER to Golgi transport [33–35].

While the molecular details of how such Rab cascades work are beginning to come to light in a small number of cases, little is known of how such activities influence endocytic recycling. In this study, we focused on the transition from early endosomes, controlled by RAB-5, to recycling endosomes, controlled by RAB-10, acting in the basolateral recycling pathway of the *C*. *elegans* intestinal epithelia. Our study shows that the downstream Rab, RAB-10, in its GTP-bound form, binds to RAB-5 GAP TBC-2 and is required for its recruitment to endosomes. Consistent with a RAB-10 to RAB-5 negative regulatory loop via TBC-2, loss of TBC-2 or RAB-10 increases association of RAB-5 with membranes, indicating abnormally high RAB-5 activation. Lack of TBC-2 also causes a dramatic morphological change in the RAB-5 labeled early endosomes. We observed accumulation of abnormally large, RAB-5-positive, pleiomorphic endosome structures, many of which displayed increased overlap with RAB-10. Thus we propose that TBC-2 can serve as a bridge in the interaction between RAB-10 and RAB-5. This model suggests that without TBC-2, RAB-5 cannot be inactivated as part of the recycling pathway, and RAB-10 endosomes cannot properly separate from RAB-5 endosomes. Our cargo localization analysis shows that in *tbc-2* mutants the recycling cargo hTAC is mostly trapped in RAB-5 positive endosomes, indicating a defect in the exit of recycling cargo from early endosomes that cannot inactivate RAB-5. Our work is consistent with, and extends, work in *C*. *elegans* neurons that independently identified a connection between RAB-10 and TBC-2 important for neuropeptide secretion [19]. Thus the biogenesis and/or cargo loading of dense-core granules appears to share mechanistic similarities with endocytic recycling. Our results are also reminiscent of a counter-current GAP cascade in *Saccharomyces cerevisiae* that is required to restrict the spatial overlap of early and late Golgi Rabs Ypt1p and Ypt32p [36].

Our study also showed that cargo transition from early endosomes to recycling endosomes requires the coordination of another regulator of the recycling pathway, BAR-domain protein AMPH-1. Like RAB-10, AMPH-1 contributes to endosomal recruitment of TBC-2. We also detected failure in proper separation of RAB-5 and RAB-10 and failure in the exit of recycling cargo from early endosomes in *amph-1* mutants, although the endosomes did not appear as grossly enlarged as in *tbc-2* mutants. The AMPH-1 BAR domain binds directly to PI(4,5)P2 enriched membranes, can potentially sense membrane curvature, and can promote tubule formation [29]. An interesting possibility is that AMPH-1 derived membrane tubules could be directly involved in cargo transfer. Our previous work also showed that AMPH-1 binds to RME-1, a later acting player in the basolateral recycling pathway, potentially acting to coordinate early and late aspects of recycling [29].

Our current study delineated distinct regions of TBC-2 bound by RAB-10 and AMPH-1. Combined with our previous work showing a connection of CED-10/Rac1 to TBC-2 and recycling [27], our observations indicate that TBC-2 is a key feedback regulator of RAB-5, acting as a molecular nexus that integrates signals from recycling endosome regulators RAB-10, AMPH-1, and CED-10. The correct localization of peripheral membrane proteins is often maintained by multiple weak physical interactions, perhaps to more precisely position such proteins at points where multiple binding partners converge, a concept sometimes called coincidence sensing. Precise recruitment of TBC-2 to endosomes during recycling is likely to be quite important in the complex process of endosomal transport, where RAB-5 activity is essential for early aspects of the pathway but needs to be deactivated for later events. Such localization mechanisms may also be easily reversible, an important characteristic in dynamic situations.

In wild-type animals we found that RAB-5-labeled endosomes and RAB-10-labeled endosomes appear as distinct puncta that show partial overlap, suggesting that only a subpopulation of RAB-5 and RAB-10 labeled endosomes is interacting at any given time. This could imply the existence of transient interactions between RAB-5 and RAB-10 labeled endosomes that function to transfer cargo, removing recycling cargo as the early endosome matures into the late endosome. Intermediates in this process could be trapped, or delayed in resolution, in *tbc-2*, *rab-10*, and *amph-1* mutants. Such transient interactions between early and recycling endosomes have been proposed in other systems, although the detailed mechanisms remain obscure [37]. Interestingly that work also indicated a BAR domain protein (Nwk) was involved in early endosome to recycling endosome transport, perhaps indicating cargo transfer via membrane tubules. More work will be required to understand the dynamic interactions between early and recycling endosomes that mediate cargo transfer.

## Materials and Methods

### General methods and strains

All *C*. *elegans* strains were derived originally from the wild-type Bristol strain N2. Worm cultures, genetic crosses, and other *C*. *elegans* husbandry were performed according to standard protocols [38]. Strains expressing transgenes were grown at 20°C. A complete list of strains used in this study can be found in S1 Table.

### Bioinformatics analyses

Secondary structures of TBC-2 protein were predicted using the Quick2D from the Bioinformatics Toolkit (Max-Planck Institute for Developmental Biology). (Web link: http://toolkit.tuebingen.mpg.de/quick2_d)

### Yeast two-hybrid analyses

The yeast two-hybrid experiments were performed according to the procedure of the DupLEX-A yeast two-hybrid system (OriGene Technologies). All two-hybrid plasmids were generated as PCR products with Gateway attB1.1 and attB2.1 sequence extensions and were introduced into the Gateway entry vector pDONR221 by BP clonase II (Invitrogen) reaction. The bait vector pEG202-Gtwy and target vector pJG4-5-Gtwy have been described previously [39]. Origene plasmid pSH18-34 (URA3, 8 ops.-LacZ) was used as a reporter in all yeast two-hybrid experiments. Constructs were introduced into the yeast strain EGY48 (MATα trp1 his3 ura3 leu2::6 LexAop-*LEU2*) included in the system. Transformants were selected on plates lacking leucine, histidine, tryptophan, and uracil and containing 2% (wt/vol) galactose/1% (wt/vol) raffinose at 30°C for 3 days and were assayed for the expression of the *LEU2* reporter. The constructs of mutated forms of TBC-2 with alanine substitution were constructed by Q5^—^Site Directed Mutagenesis Kit (New England Biolabs, Inc.) using the cDNA sequence of TBC-2 as template.

### Plasmids and transgenic strains

To construct GFP or RFP/mCherry fusion transgenes that express specifically in the worm intestine, we used a previously described *vha-6* promoter-driven vector modified with a Gateway cassette inserted at the Asp718I site just upstream of the GFP or RFP coding region [10]. The PCR products of the genes of interest were first cloned into the Gateway entry vector pDONR221 by BP reaction (Invitrogen). Then the PDONR221 plasmids carrying the sequences of interest were transferred into the intestinal expression vectors by Gateway recombination cloning, in a LR clonase II (Invitrogen) reaction, to generate N-terminal/C-terminal fusions [10]. Low-copy integrated transgenic lines for all of these plasmids were obtained by the microparticle bombardment method [40]. Transgenic strains pwEx142-144 were generated as following. Full-length TBC-2, TBC-2(P150A), and TBC-2(288-292AAAAA) was first cloned into entry vector pDONR221. pSM47 pSNX-1::tagRFP, pDONR221 containing TBC-2, or TBC-2(P150A), TBC-2(288-292AAAAA), pCM1.36-TBB-2 3'-UTR was inserted into the pCFJ1001 vector via multi-site LR reaction (Gateway LR Clonase II Plus Enzyme by Life Technologies). Rescue plasmids pCFJ1001::pSNX-1::tagRFP::TBC-2 (full length, P150A, or 288-292AAAAA) (10 ng/ul), pCFJ601 (50 ng/ul) and pmyo-2::GFP (coinjection marker) (10 ng/ul) were microinjected and resulting extrachromosomal arrays were used in this study [41].

For yeast two-hybrid analysis pEG202-RAB-10(Q68L), pEG202-AMPH-1(SH3), and pJG4-5-TBC-2 were constructed by gateway cloning as described previously [10,29]. For GST pull-down experiments *rab-10(Q68L)* and *tbc-2* cDNA clones were transferred to in-house modified pcDNA3.1 (+) (Invitrogen) vectors containing 2xHA or 3xFLAG epitope tags and a Gateway cassette (Invitrogen) as described previously [11].

### Microscopy and image analysis

Live worms were mounted on 2% agarose pads with 10mM levamisole as described previously [39]. Multiwavelength fluorescence colocalization images were obtained using an Axio Imager.Z1 microscope (Carl Zeiss Microimaging) equipped with a YOKOGAWA CSU-X1 spinning disk, Photometrics Evolve 512 EMCCD camera, captured using Metamorph software (Universal Imaging), and then deconvolved using AutoQuant X5 (AutoQuant Imaging). Images taken in the DAPI channel were used to identify broad-spectrum intestinal autofluorescence caused by lipofuscin-positive lysosome-like organelles [42,43]. Quantification of colocalization images was done using the open source Fiji (Image J) software [44]. GFP/RFP colocalization experiments were performed on L4 larvae expressing GFP and RFP markers as previously described. To obtain images of GFP fluorescence without interference from autofluorescence, we used argon 488-nm excitation and the spectral fingerprinting function of the Zeiss LSM710 Meta confocal microscope system (Carl Zeiss Microimaging). Quantification of images was performed with Metamorph Version 6.3r2 (Universal Imaging).

### Membrane fractionation

Worms expressing intestinal GFP-RAB-5 in wild-type, *tbc-2(tm2241)*, *rab-10(q373)* and *amph-1(tm1060)* genetic backgrounds were synchronized and cultured on NGM. Mixed stage worms were washed off with M9 buffer, pelleted and resuspended in 500μl of lysis buffer (50 mM Tris-HCL PH 8.0, 20% Sucrose, 10% Glycerol, 2 mM DTT and protease inhibitors). The worms are then disrupted using a Mini-Beadbeater-16 (BioSpec Products). Carcasses and nuclei were removed by centrifugation at 1000g for 5 min at 4°C. 200 μl of the postnuclear lysate was centrifuged at 100,000g for 1h. Pellets were reconstituted in the same volume of lysis buffer as that recovered as supernatant.

### *C*. *elegans* western analysis

Worms expressing intestinal GFP-TBC-2 in wild-type, *rab-10(ok1494)*, and *amph-1(tm1060)* genetic backgrounds were synchronized and cultured on NGM plates. 50 young adult animals of each genotype were handpicked into 10 μl of lysis buffer (100 mM Tris pH 6.8, 8% SDS, 20 mM β-mercaptoethanol) and boiled at 100°C for 10 min. Extracted worm proteins were separated by 10% SDS-PAGE and blotted to nitrocellulose. After blocking, the blot was probed with HRP-conjugated anti-GFP antibody.

### GST pull-down analysis

rab-10(Q68L) and tbc-2 cDNA clones were transferred to in-house modified pcDNA3.1 (+) (Invitrogen) vectors containing 2xHA or 3xFLAG epitope tags and a Gateway cassette (Invitrogen) for *in vitro* transcription/translation experiments using the TNT-coupled transcription-translation system (Promega). Full length GST and GST-AMPH-1 was expressed and purified as previously described [29]. Eluted proteins were separated by ExpressPlus PAGE (4–20%) (GenScript), blotted to nitrocellulose, and stained with Ponceau S to detect GST fusion proteins. After blocking, the blot was probed with anti-HA (16B12) antibody and anti-FLAG M2-Peroxidase antibody (Sigma-Aldrich).

## Supporting Information

S1 Fig Mutated forms of TBC-2 (P150A, P153A, or R155A) which are incapable of binding to AMPH-1, can still interact with RAB-10(Q68L). (A) RAB-10(Q68L) was expressed in a yeast reporter strain as a fusion to the DNA-binding domain of LexA (bait). Mutated forms of TBC-2 (P150A, P153A or R155A) were expressed in the same yeast cells as fusions to the B42 transcriptional activation domain (prey). Interaction between bait and prey was assayed by complementation of leucine auxotrophy (LEU2 growth assay). Colonies were diluted in liquid and spotted on solid growth medium directly (1X) or after further dilution (0.1X).
(TIF)

S2 Fig TBC-2 colocalizes partially with RAB-5 and RAB-7, and does not colocalize with EHBP-1. All images are from deconvolved 3D confocal image stacks acquired in intact living animals expressing GFP- and RFP-tagged proteins specifically in intestinal epithelial cells. (A-A'') GFP-TBC-2 colocalizes partially with tagRFP-RAB-5 on punctate endosomal structures. (B-B'') GFP-TBC-2 colocalizes partially with tagRFP-RAB-7. (C-C'') GFP-TBC-2 displays little colocalization with EHBP-1-MC. In each image autofluorescent lysosome-like organelles appears in blue in all three channels, whereas GFP appears only in the green channel and RFP shows up only in the red channel. Signals observed in the green or red channels that do not overlap with signals in the blue channel are considered bona fide GFP or RFP signals respectively. (Scale bar: 10 μm) (D) Pearson's correlation coefficient for colocalization of GFP-TBC-2 with tagRFP-RAB-5, tagRFP-RAB-7, and EHBP-1-MC. n = 6. Error bars represent SEM.
(TIF)

S3 Fig AMPH-1 colocalizes with RAB-10 on endosomes and AMPH-1 interacts physically with RAB-10. All images are from deconvolved 3D confocal image stacks acquired in intact living animals expressing GFP- and RFP-tagged proteins specifically in intestinal epithelial cells. (A-A'') AMPH-1-GFP colocalizes well with tagRFP-RAB-10. Arrowheads indicate endosomes labeled by both AMPH-1-GFP and tagRFP-RAB-10. (A‴) Magnified image of A'' is designated by rectangular outline. In each image autofluorescent lysosome-like organelles appear in blue in all three channels, whereas GFP appears only in the green channel and RFP shows up only in the red channel. Signals observed in the green or red channels that do not overlap with signals in the blue channel are considered *bona fide* GFP or RFP signals respectively. (Scale bar: 10 μm) (B) Pearson's correlation coefficient for colocalization of AMPH-1-GFP with tagRFP-RAB-10. n = 6. Error bars represent SEM. (C) Glutathione beads loaded with recombinant GST or GST-AMPH-1 were incubated with *in vitro*-translated target proteins HA-RAB-10(Q68L) and/or FLAG-TBC-2. Bound proteins were analyzed by western blotting with anti-HA and anti-FLAG antibodies. Counting from the left, the third lane contains a mix of 5% HA-RAB-10(Q68L) and 1% FLAG-TBC-2 inputs, the fourth lane contains 10% HA-RAB-10 input, and the sixth lane contains 10% FLAG-TBC-2 input. The lower panel shows equivalent loading of bait GST fusion proteins as visualized by Ponceau S staining of the blot prior to antibody incubation.
(TIF)

S4 Fig Loss of RAB-10 results in increased AMPH-1-GFP intensity. All images are from deconvolved 3D confocal image stacks acquired in intact living animals expressing GFP-tagged protein. (A-B) Representative confocal images of the worm intestine expressing AMPH-1-GFP. Loss of RAB-10 caused increased AMPH-1-GFP intensity on puncta and tubules in the worm intestinal cells. (Scale bar: 10 μm) (C) Quantification of the average puncta intensity of AMPH-1-GFP. In each image autofluorescent lysosome-like organelles appear in blue in both channels, whereas GFP appears only in the green channel. Signals observed in the green channels that do not overlap with signals in the blue channel are considered *bona fide* GFP signals. (n = 18 each, 6 animals of each genotype sampled in three different regions of each intestine.) Error bars represent SEM. ***P<0.001(student's t test).
(TIF)

S5 Fig *amph-1* mutant animals display increased RAB-5 intensity. All images were collected from living intact adult animals expressing intestine-specific GFP-RAB-5. (A-B) Representative confocal images of GFP-RAB-5 in wild-type and *amph-1(tm1060)* mutant backgrounds are shown. The intensity of GFP-RAB-5 puncta is increased in *amph-1(tm1060)* mutant background. (Scale bar: 10 μm) (C) Quantification of the average puncta intensity of GFP-RAB-5 labeled structures. (n = 18 each, 6 animals of each genotype sampled in three different regions of each intestine.) Error bars represent SEM. ***P<0.001(student's t test).
(TIF)

S1 Table Strain list. Summary of the transgenic and mutant strains used in this work.
(DOCX)
