# Supplementary figures and images for: Basolateral Endocytic Recycling Requires RAB-10 and AMPH-1 Mediated Recruitment of RAB-5 GAP TBC-2 to Endosomes
Source: PLoS Genet. 2015 Sep 22;11(9):e1005514. doi: 10.1371/journal.pgen.1005514 (PMC4578947; doi:10.1371/journal.pgen.1005514)

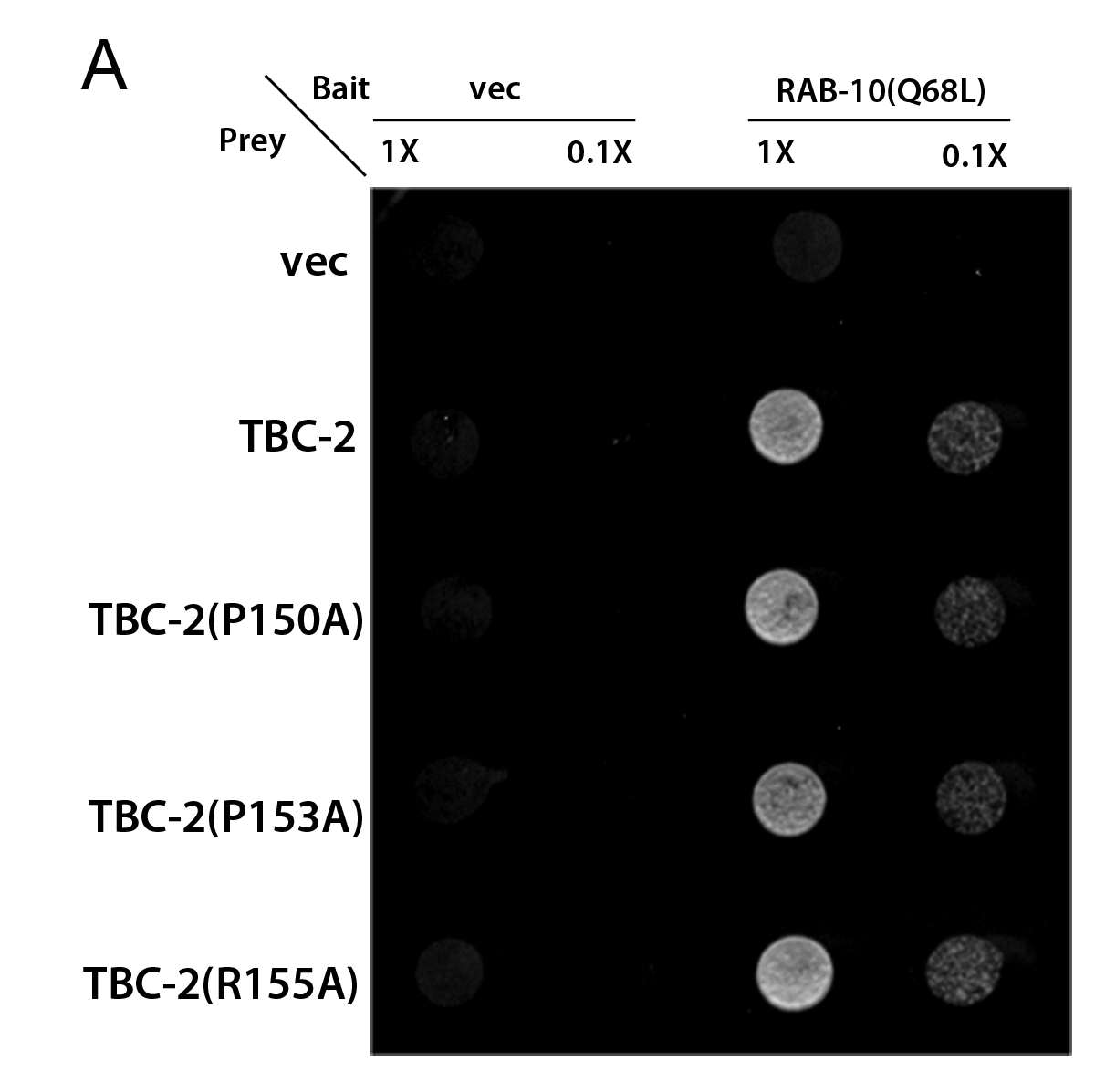

Supplement: S1 Fig — (A) RAB-10(Q68L) was expressed in a yeast reporter strain as a fusion to the DNA-binding domain of LexA (bait). Mutated forms of TBC-2 (P150A, P153A or R155A) were expressed in the same yeast cells as fusions to the B42 transcriptional activation domain (prey). Interaction between bait and prey was assayed by complementation of leucine auxotrophy (LEU2 growth assay). Colonies were diluted in liquid and spotted on solid growth medium directly (1X) or after further dilution (0.1X). (TIF) [file pgen.1005514.s001.tif]

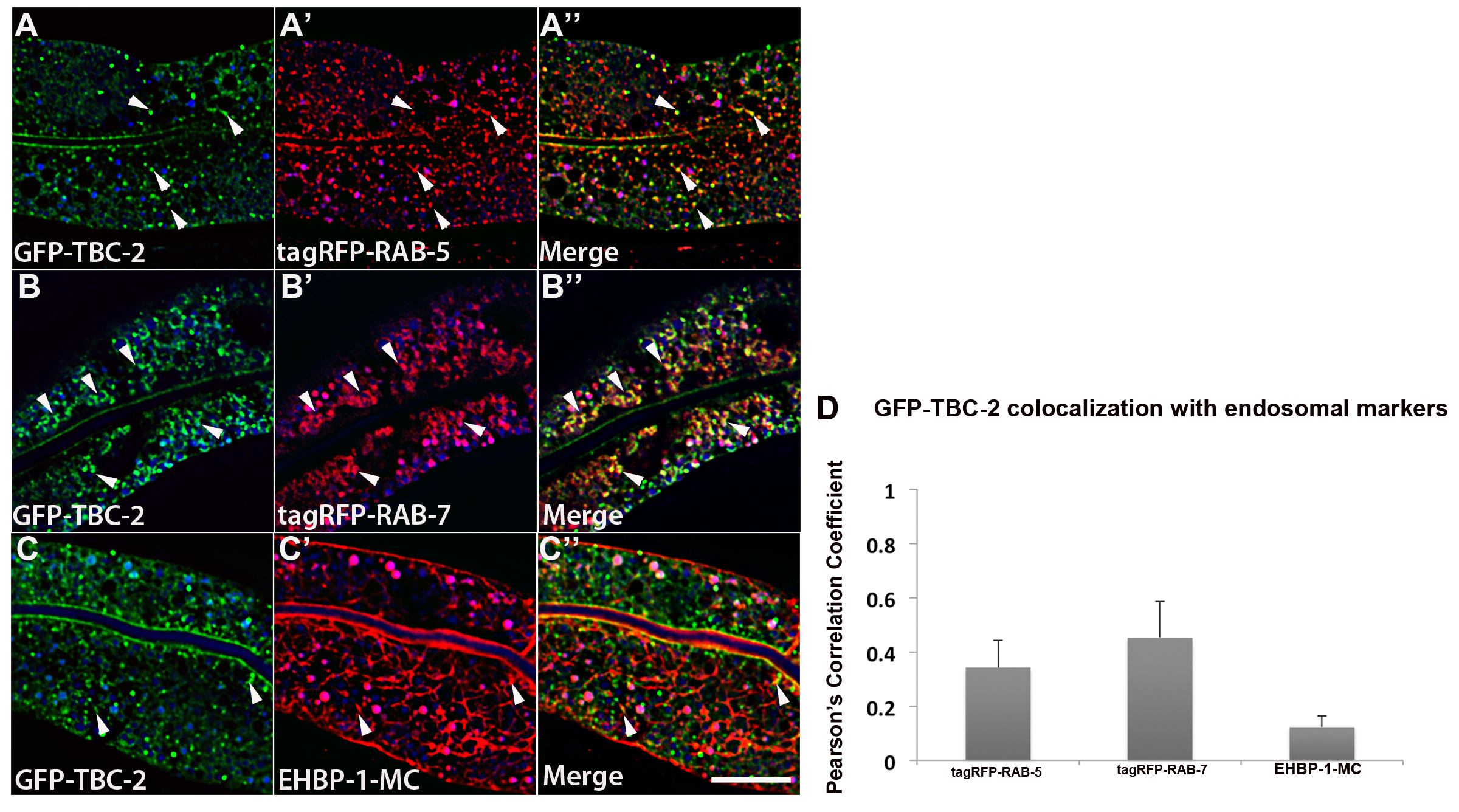

Supplement: S2 Fig — All images are from deconvolved 3D confocal image stacks acquired in intact living animals expressing GFP- and RFP-tagged proteins specifically in intestinal epithelial cells. (A-A'') GFP-TBC-2 colocalizes partially with tagRFP-RAB-5 on punctate endosomal structures. (B-B'') GFP-TBC-2 colocalizes partially with tagRFP-RAB-7. (C-C'') GFP-TBC-2 displays little colocalization with EHBP-1-MC. In each image autofluorescent lysosome-like organelles appears in blue in all three channels, whereas GFP appears only in the green channel and RFP shows up only in the red channel. Signals observed in the green or red channels that do not overlap with signals in the blue channel are considered bona fide GFP or RFP signals respectively. (Scale bar: 10 μm) (D) Pearson's correlation coefficient for colocalization of GFP-TBC-2 with tagRFP-RAB-5, tagRFP-RAB-7, and EHBP-1-MC. n = 6. Error bars represent SEM. (TIF) [file pgen.1005514.s002.tif]

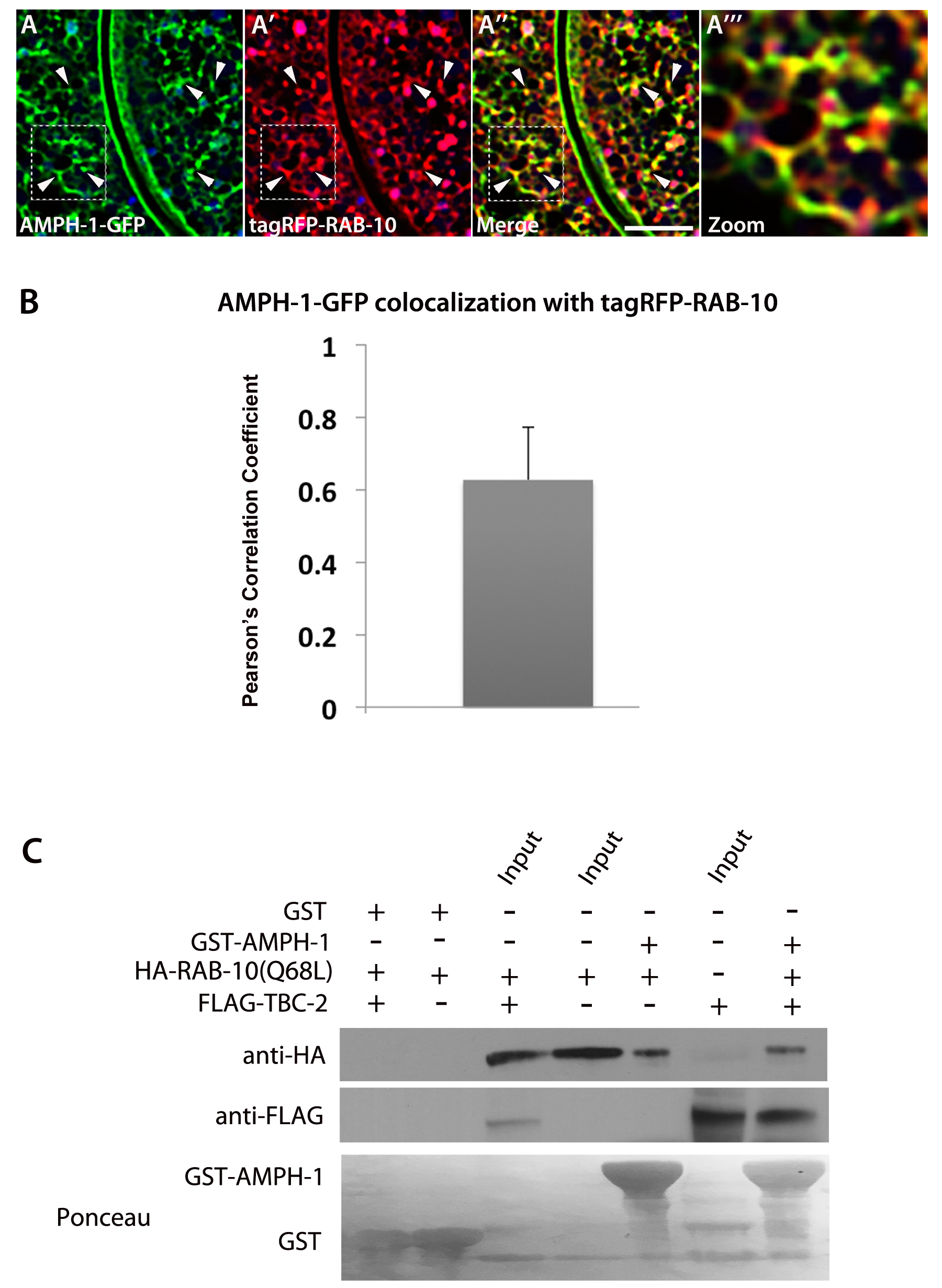

Supplement: S3 Fig — All images are from deconvolved 3D confocal image stacks acquired in intact living animals expressing GFP- and RFP-tagged proteins specifically in intestinal epithelial cells. (A-A'') AMPH-1-GFP colocalizes well with tagRFP-RAB-10. Arrowheads indicate endosomes labeled by both AMPH-1-GFP and tagRFP-RAB-10. (A‴) Magnified image of A'' is designated by rectangular outline. In each image autofluorescent lysosome-like organelles appear in blue in all three channels, whereas GFP appears only in the green channel and RFP shows up only in the red channel. Signals observed in the green or red channels that do not overlap with signals in the blue channel are considered bona fide GFP or RFP signals respectively. (Scale bar: 10 μm) (B) Pearson's correlation coefficient for colocalization of AMPH-1-GFP with tagRFP-RAB-10. n = 6. Error bars represent SEM. (C) Glutathione beads loaded with recombinant GST or GST-AMPH-1 were incubated with in vitro-translated target proteins HA-RAB-10(Q68L) and/or FLAG-TBC-2. Bound proteins were analyzed by western blotting with anti-HA and anti-FLAG antibodies. Counting from the left, the third lane contains a mix of 5% HA-RAB-10(Q68L) and 1% FLAG-TBC-2 inputs, the fourth lane contains 10% HA-RAB-10 input, and the sixth lane contains 10% FLAG-TBC-2 input. The lower panel shows equivalent loading of bait GST fusion proteins as visualized by Ponceau S staining of the blot prior to antibody incubation. (TIF) [file pgen.1005514.s003.tif]

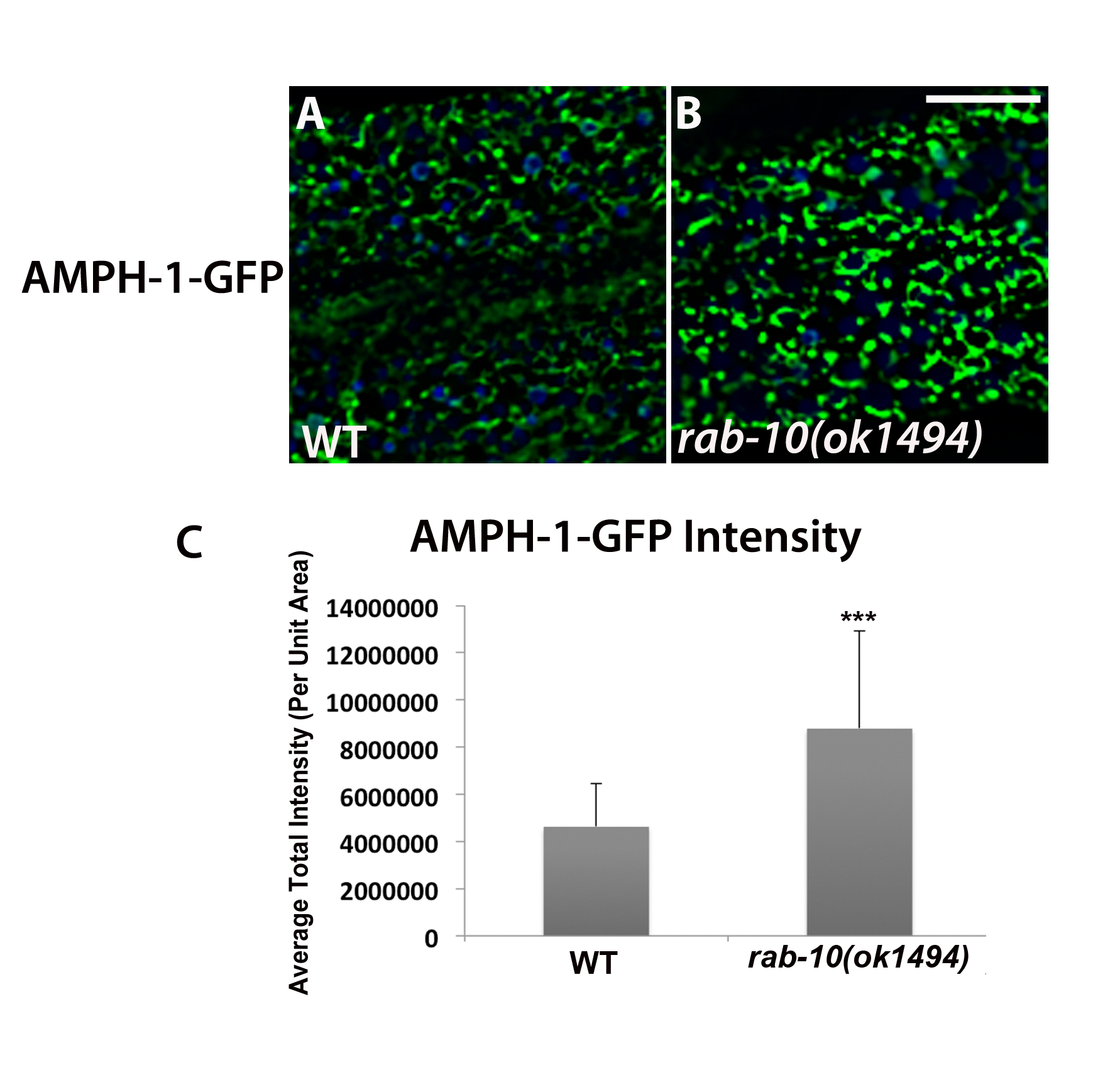

Supplement: S4 Fig — All images are from deconvolved 3D confocal image stacks acquired in intact living animals expressing GFP-tagged protein. (A-B) Representative confocal images of the worm intestine expressing AMPH-1-GFP. Loss of RAB-10 caused increased AMPH-1-GFP intensity on puncta and tubules in the worm intestinal cells. (Scale bar: 10 μm) (C) Quantification of the average puncta intensity of AMPH-1-GFP. In each image autofluorescent lysosome-like organelles appear in blue in both channels, whereas GFP appears only in the green channel. Signals observed in the green channels that do not overlap with signals in the blue channel are considered bona fide GFP signals. (n = 18 each, 6 animals of each genotype sampled in three different regions of each intestine.) Error bars represent SEM. ***P<0.001(student's t test). (TIF) [file pgen.1005514.s004.tif]

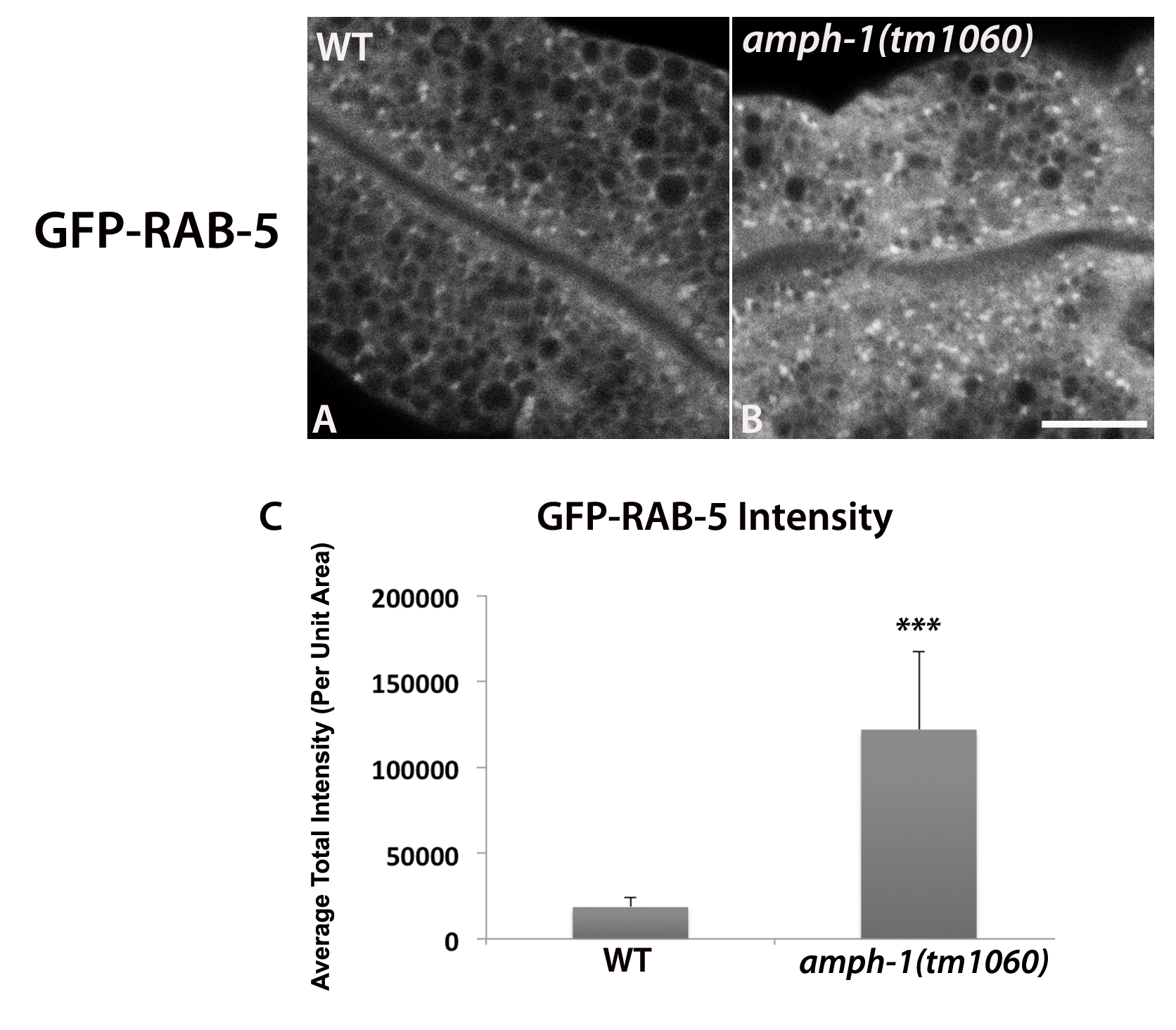

Supplement: S5 Fig — All images were collected from living intact adult animals expressing intestine-specific GFP-RAB-5. (A-B) Representative confocal images of GFP-RAB-5 in wild-type and amph-1(tm1060) mutant backgrounds are shown. The intensity of GFP-RAB-5 puncta is increased in amph-1(tm1060) mutant background. (Scale bar: 10 μm) (C) Quantification of the average puncta intensity of GFP-RAB-5 labeled structures. (n = 18 each, 6 animals of each genotype sampled in three different regions of each intestine.) Error bars represent SEM. ***P<0.001(student's t test). (TIF) [file pgen.1005514.s005.tif]
