# Supplementary material for: Basolateral Endocytic Recycling Requires RAB-10 and AMPH-1 Mediated Recruitment of RAB-5 GAP TBC-2 to Endosomes
Source: PLoS Genet. 2015 Sep 22;11(9):e1005514. doi: 10.1371/journal.pgen.1005514 (PMC4578947; doi:10.1371/journal.pgen.1005514)
Supplement: S1 Table — Summary of the transgenic and mutant strains used in this work. (DOCX) [file pgen.1005514.s006.docx]

**Table S1. Transgenic and mutant strains used in this study**

*pwIs72[Pvha-6::GFP::RAB-5]* [10]

*pwIs112[Pvha-6::hTAC::GFP]* [10]

*pwIs206[Pvha-6::GFP::RAB-10]* [10]

*pwIs630 Pvha-6-AMPH-1-GFP* [29]

*pwIs717[Pvha-6::hTfR::GFP]* [27]

*pwIs728[Pvha6::CNT-1::mCherry]* [11]

*pwIs846[Pvha-6::RFP::RAB-5]* [27*]*

*pwIs849[Pvha-6::RFP::RAB-7]* [45]

*pwIs883[Pvha-6::EHBP-1::mCherry]* [27]

*pwIs957[Pvha-6::RFP::RAB-10]* [27]

*pwIs1195[Pvha-6::GFP::RAB-10(Q68L)]* (this work)

*pwEx142[Psnx-1::RFP::TBC-2]* (this work)

*pwEx143[Psnx-1::RFP::TBC-2(288-292 AAAAA)]* (this work)

*pwEx144[Psnx-1::RFP::TBC-2(P150A)]* (this work)

*vhIs12[Pvha-6::GFP::TBC-2]* [25]

*rab-10(ok1494)* [11]

*rab-10(q373)*[10]

*tbc-2(tm2241)* [25]

*amph-1(tm1060)* [29]

45. Gleason RJ, Akintobi AM, Grant BD, Padgett RW (2014) BMP signaling requires retromer-dependent recycling of the type I receptor. Proc Natl Acad Sci U S A 111: 2578-2583.
